# Accelerated DNA methylation age and medication use among African Americans

**DOI:** 10.18632/aging.203115

**Published:** 2021-06-03

**Authors:** Minjung Kho, Yi Zhe Wang, Dima Chaar, Wei Zhao, Scott M. Ratliff, Thomas H. Mosley, Patricia A. Peyser, Sharon L.R. Kardia, Jennifer A. Smith

**Affiliations:** 1 Department of Epidemiology, School of Public Health, University of Michigan, Ann Arbor, MI 48109, USA; 2 Memory Impairment and Neurodegenerative Dementia (MIND) Center, University of Mississippi Medical Center, Jackson, MS 39216, USA

**Keywords:** DNA methylation, epigenetic clock, methylation age, medication use, African Americans

## Abstract

DNA methylation age acceleration, the discrepancy between epigenetic age and chronological age, is associated with mortality and chronic diseases, including diabetes, hypertension, and hyperlipidemia. In this study, we investigate whether medications commonly used to treat these diseases in 15 drug categories are associated with four epigenetic age acceleration measures: HorvathAge acceleration (HorvathAA), HannumAge acceleration (HannumAA), PhenoAge acceleration, and GrimAge acceleration (GrimAA) using cross-sectional (Phase 1, N=1,100) and longitudinal (Phases 1 and 2, N=266) data from African Americans in the Genetic Epidemiology Network of Arteriopathy (GENOA) study. In cross-sectional analyses, the use of calcium channel blockers was associated with 1.27 years lower HannumAA after adjusting for covariates including hypertension (p=0.001). Longitudinal analyses showed that, compared to those who never used antihypertensives, those who started to take antihypertensives after Phase 1 had a 0.97-year decrease in GrimAA (p=0.007). In addition, compared to those who never used NSAID analgesics, those who started to take them after Phase 1 had a 2.61-year increase in HorvathAA (p=0.0005). Our study demonstrates that three commonly used medications are associated with DNAm age acceleration in African Americans and sheds light on the potential epigenetic effects of pharmaceuticals on aging at the cellular level.

## INTRODUCTION

Aging is a major risk factor for most chronic diseases, including cancer, cardiovascular disease (CVD), type 2 diabetes, and neurodegenerative diseases [1], all of which have become increasingly prevalent as the US population ages [2–4]. In 2015-2016, nearly 70% of adults aged 40-79 in the United States had used at least one prescription drug in the past 30 days to manage or prevent health conditions [5]. For those aged 60 and over, the most commonly used drug categories include antidiabetic medications, beta-blockers for high blood pressure and heart disease, and antilipidemics [5]. These medications, which are considered promising drug candidates for “anti-aging medicine” [6, 7], as well as nonsteroidal anti-inflammatory drugs (NSAIDs), play an important role in reducing aging-related pathology through reduction of inflammation in CVD. However, some of these drugs also have adverse effects on health [8–11].

DNA methylation (DNAm) is an epigenetic mechanism that modifies the expression of genes and may be influenced by environmental exposures, including diet [12], medication use [13], toxicants [14], lifestyle factors [15], and disease exposure across the human lifespan [16]. Several recently developed DNAm clocks using weighted averages of methylation levels at specific CpG sites have been trained on various metrics such as age, sex, aging-related clinical measurements, plasma proteins, and/or smoking [17–20]. The importance of DNAm age acceleration – the discrepancy between epigenetic age and chronological age – has been spotlighted because of its association with multiple aging-related chronic diseases and mortality risks [21, 22].

The relationship between epigenetic age acceleration and demographic factors, lifestyle factors, and health-related traits has been actively investigated to better understand how risk factors contribute to aging at the cellular level [21, 23, 24]. However, limited studies have evaluated the influence of commonly used pharmaceuticals on DNAm age acceleration. Since taking specific pharmaceuticals (e.g., antidiabetics, antihypertensives, or antilipidemics) reduces the severity or risk of developing aging-related diseases, it is possible that using these medications may also lead to decreases in DNAm age acceleration through deceleration of the biological aging process caused by relevant health conditions. On the other hand, recent evidence suggests that exposure to commonly used pharmaceuticals may also contribute to age-related chronic diseases, including CVD, cancer, neurological disorders, and diabetes, through epigenetic side effects [25, 26]. Thus, it is critical to comprehensively investigate how commonly used pharmaceuticals are associated with biological aging and examine which drugs may have preventive or adverse effects on DNAm age acceleration.

Gao et al. [27] found that antihypertensive medication use increased a measure of DNAm age acceleration developed by Horvath et al. [18]. Antihypertensive medication use also increased the rate of change in this age acceleration measure over approximately 4 years among older males of European ancestry from the Normative Aging Study (NAS) after adjusting for potential covariates, including hypertension. However, the sample size was relatively small (N=546) and included only male subjects, and the follow-up duration may not have been long enough to adequately capture the preventive effects of antihypertensive medication on DNAm age acceleration. Given that the aging process may differ by sex and race/ethnicity [28, 29], studies are needed to comprehensively evaluate the effects of commonly used medications on DNAm age acceleration using multiple DNAm aging clocks in other populations. This is particularly important in African Americans, who have the highest burden of hypertension in the United States [30].

The aim of this study is to investigate the cross-sectional and longitudinal associations between commonly used medications and four DNAm age acceleration measures in African Americans from the Genetic Epidemiology Network of Arteriopathy (GENOA) study.

## RESULTS

### Participant characteristics

Phase 1 characteristics for GENOA participants are described in Table 1. Mean DNAm age estimates (ranging from 44.2 to 54.3 years) were lower than chronological age (mean = 57.0 years). DNAm age acceleration measures are also reported in Table 1 with PhenoAge acceleration (PhenoAA) being the highest (mean = 0.38 years) followed by HannumAge acceleration (HannumAA, mean = 0.15 years), HorvathAge acceleration (HorvathAA, mean = 0.14 years), and GrimAge acceleration (GrimAA, mean = 0.11 years). In the subset of the sample used for longitudinal analyses, the participants at Phase 2 were about 5.4 years older than at Phase 1 (mean age = 54.0 years at Phase 1 and 59.4 years at Phase 2).

**Table 1:** Descriptive statistics of study participants from the genetic epidemiology network of arteriopathy (GENOA).

	**Cross-Sectional (N=1,100)**	**Longitudinal (N=266)**	
	**Phase 1**	**Phase 1**	**Phase 2**
Chronological age (years)	57.0 (10.5)	54.0 (9.8)	59.4 (9.4)
HorvathAge (years)	53.9 (10.0)	51.2 (9.2)	55.1 (9.2)
HannumAge (years)	47.7 (10.8)	44.3 (9.8)	49.1 10.0)
PhenoAge (years)	44.2 (12.7)	40.2 (11.6)	44.7 (11.7)
GrimAge (years)	54.3 (9.5)	52.0 (9.1)	55.4 (9.2)
HorvathAA(years)	0.14 (5.1)	-0.063 (5.1)	-0.56 (5.5)
HannumAA (years)	0.15 (4.8)	-0.47 (4.7)	-0.59 (5.3)
PhenoAA (years)	0.38 (7.2)	-0.53 (6.8)	-1.46 (6.3)
GrimAA (years)	0.11 (5.0)	0.23 (4.6)	-0.58 (4.6)
Female	781 (71.0%)	189 (71.0%)	
Smoking			
Never	666 (60.6%)	160 (60.2%)	
Former	255 (23.2%)	63 (23.7%)	
Current	179 (16.3%)	43 (16.2%)	
Education			
Less than high school	374 (34.0%)	80 (30.1%)	
HS/GED	292 (26.6%)	71 (26.7%)	
At least some college	434 (39.4%)	115 (43.2%)	
Continuous drinks/week	0.67 (2.7)	0.74 (2.8)	0.52 (1.7)
Body Mass Index (kg/m^2^)	31.2 (6.5)	31.5 (6.7)	32.0 (6.8)
Hypertension	776 (70.5%)	174 (65.4%)	203 (76.3%)
Diabetes	216 (19.6%)	44 (16.5%)	69 (25.9%)
Stroke	40 (3.7%)	9 (3.4%)	13 (4.9%)
Coronary heart disease	47 (4.3%)	8 (3.0%)	13 (4.9%)
Lipids			
High-density lipoprotein (mg/dL)	55.2 (17.9)	55.5 (17.9)	58.0 (18.5)
Triglycerides (mg/dL)	147.6 (98.8)	150.3 (98.7)	117.8 (78.1)
Low-density lipoprotein (mg/dL)	120.1 (43.1)	118.7 (41.4)	118.8 (38.2)

HorvathAA: DNAm HorvathAge acceleration; HannumAA: DNAm HannumAge acceleration; PhenoAA: DNAm PhenoAge acceleration; GrimAA: DNAm GrimAge acceleration; HS/GED: High School/General Education Development.

Mean (SD) or N (%) is displayed.

The sample size for PhenoAge and GrimAge was 1,099.

Supplementary Table 1 shows medication use categorized by the first two digits of the Medi-Span Therapeutic Classification (MTC) code. Although diuretics, calcium channel blockers, and beta blockers are often prescribed as antihypertensive medications, they have different MTC codes than antihypertensives because they are used for other conditions as well. Drug categories with N < 30 were excluded. For the three major drug categories (antihypertensives, antihyperlipidemics, and diabetes medications), sub-drug categories were also included. The number of participants taking each drug was highest for diuretics (N=377) followed by antihypertensives (N=321), calcium channel blockers (N=221), and diabetes medications (N=164). Supplementary Table 2 shows the p-value of the associations between medication categories. Diuretic medication use was associated with most of the other medication categories, especially with the use of other antihypertensive medications (significant P range: 4.7 x 10^-11^ – 0.03). Also, the use of renin-angiotensin-aldosterone system (RAAS) inhibitors was associated with diabetes medication (P< 1 x 10^-10^). Both non-narcotic analgesic use and NSAID analgesic use were also significantly associated with several other medication use categories (significant P range: 9.6 x 10^-10^ – 0.03).

### Correlation among DNAm age estimators

The correlations between chronological age and DNAm age estimated by the four epigenetic clocks are presented in Supplementary Figure 1. As expected, chronological age was positively and significantly correlated with HorvathAge (Pearson correlation coefficient r = 0.86), HannumAge (r = 0.90), PhenoAge (r = 0.82), and GrimAge (r = 0.85). The Pearson correlation matrix of DNAm age acceleration, both before and after adjustment for white blood cell proportions, is shown in Supplementary Figure 2. The correlations range from 0.18 (GrimAA and HorvathAA) to 0.58 (HorvathAA and HannumAA). GrimAA was the least correlated with other AA measures.

### Associations between medication use and DNAm age acceleration

We first fit a single multivariable model with all drug categories (N=15) to examine the unique contribution of each medication to each DNAm age acceleration measure using cross-sectional data from Phase 1 (N=1,100, Table 2). We were interested in associations that were significant at a nominal level (P<0.05) as well as at a Bonferroni-corrected significance threshold (P<.05/15 = 0.003). Model 1 adjusted for age and sex, and Model 2 also included body mass index (BMI), smoking, education, and alcohol consumption. Model 3 additionally adjusted for white blood cell proportions. Calcium channel blockers were independently associated with a 1.16-year lower HannumAA at the Bonferroni-adjusted significance threshold after adjusting for sex, BMI, smoking, education, and alcohol consumption (P=0.001, Model 2). The association remained significant after further adjustment for white blood cell proportions (Model 3).

**Table 2:** Association of DNA methylation age acceleration with drug categories using multivariable models.

	**HorvathAA (N=1,100)**							**HannumAA (N=1,100)**					
	**Model 1**		**Model 2**		**Model 3**			**Model 1**		**Model 2**		**Model 3**	
	**Beta**	**P-value**	**Beta**	**P-value**	**Beta**	**P-value**		**Beta**	**P-value**	**Beta**	**P-value**	**Beta**	**P-value**
Diuretics	0.37	0.281	0.36	0.311	0.31	0.354		0.32	0.313	0.31	0.335	0.24	0.425
Calcium channel blockers	-0.83	**0.030**	-0.86	**0.025**	-0.82	**0.027**		-1.00	**0.005**	-1.16	0.001*	-1.12	0.001*
Beta blockers	0.02	0.974	0.01	0.985	-0.02	0.971		-0.92	**0.047**	-0.90	0.052	-0.95	**0.028**
Alpha blockers	-1.20	0.073	-1.10	0.099	-0.95	0.139		-0.11	0.862	-0.07	0.913	0.16	0.776
Sympatholytics	0.002	0.998	-0.13	0.850	-0.26	0.697		0.18	0.775	-0.06	0.920	-0.30	0.618
RAAS inhibitors	0.48	0.236	0.38	0.347	0.31	0.429		0.10	0.794	0.03	0.939	-0.05	0.877
Statins	-0.003	0.997	0.13	0.860	0.14	0.843		0.04	0.953	0.23	0.733	0.30	0.636
Sulfonylureas	1.23	**0.035**	1.18	**0.044**	1.15	**0.041**		0.55	0.304	0.47	0.389	0.53	0.301
Insulins	1.24	**0.040**	1.04	0.085	0.93	0.110		1.42	**0.012**	1.38	**0.014**	1.10	**0.038**
Non-narcotic analgesics	-0.17	0.725	-0.37	0.440	0.10	0.823		-0.48	0.277	-0.53	0.232	0.01	0.972
NSAID analgesics	-0.83	0.079	-0.92	0.050	-1.04	**0.022**		-0.70	0.105	-0.68	0.117	-0.78	0.056
Antidepressants	0.68	0.312	0.72	0.282	0.72	0.259		0.44	0.478	0.43	0.486	0.48	0.410
Antihistamines	0.21	0.770	0.22	0.759	0.11	0.875		0.20	0.758	0.27	0.682	0.14	0.817
Antianxiety medications	0.22	0.762	0.23	0.760	-0.02	0.975		0.19	0.781	0.24	0.726	0.13	0.838
Narcotic analgesics	0.39	0.668	0.23	0.803	0.00	0.998		-0.09	0.915	-0.35	0.680	-0.57	0.473
	**PhenoAA (N=1,099)**							**GrimAA (N=1,099)**					
	**Model 1**		**Model 2**		**Model 3**			**Model 1**		**Model 2**		**Model 3**	
	**Beta**	**P-value**	**Beta**	**P-value**	**Beta**	**P-value**		**Beta**	**P-value**	**Beta**	**P-value**	**Beta**	**P-value**
Diuretics	0.67	0.159	0.66	0.172	0.70	0.118		-0.08	0.792	0.21	0.407	0.30	0.217
Calcium channel blockers	-0.07	0.897	-0.27	0.608	-0.19	0.697		0.63	0.071	0.32	0.252	0.37	0.165
Beta blockers	0.12	0.862	0.08	0.908	-0.13	0.836		-0.23	0.614	-0.20	0.590	-0.29	0.393
Alpha blockers	-1.79	0.053	-1.77	0.054	-2.04	**0.017**		0.55	0.366	0.47	0.339	0.19	0.682
Sympatholytics	0.14	0.882	-0.05	0.958	-0.58	0.519		0.74	0.245	0.47	0.360	0.20	0.671
RAAS inhibitors	0.22	0.697	0.13	0.807	-0.19	0.712		0.62	0.094	0.67	**0.025**	0.50	0.075
Statins	0.45	0.657	0.47	0.643	-0.24	0.795		1.74	**0.009**	1.26	**0.019**	0.79	0.118
Sulfonylureas	1.70	**0.035**	1.65	**0.041**	1.74	**0.021**		0.66	0.214	0.57	0.187	0.50	0.213
Insulins	2.11	**0.011**	2.06	**0.013**	1.36	0.080		0.78	0.153	1.12	**0.012**	0.83	**0.048**
Non-narcotic analgesics	0.32	0.625	0.21	0.745	0.16	0.794		0.74	0.090	0.75	**0.032**	0.46	0.172
NSAID analgesics	-1.01	0.117	-0.98	0.128	-1.12	0.062		-0.31	0.464	0.09	0.794	0.08	0.803
Antidepressants	1.78	0.054	1.74	0.057	1.78	**0.037**		1.29	**0.034**	0.88	0.070	0.89	0.054
Antihistamines	-0.56	0.569	-0.39	0.690	-0.25	0.782		-1.16	0.072	-0.54	0.296	-0.42	0.392
Antianxiety medications	-0.80	0.436	-0.74	0.471	-0.71	0.455		0.49	0.470	0.31	0.574	0.23	0.657
Narcotic analgesics	-0.08	0.947	-0.46	0.709	-0.44	0.701		0.52	0.533	-0.23	0.728	-0.26	0.674

GrimAA: DNA methylation GrimAge acceleration; HannumAA: DNA methylations HannumAge acceleration; HorvathAA: DNA methylations HorvathAge acceleration; NSAID: nonsteroidal anti-inflammatory drug; PhenoAA: DNA methylations PhenoAge acceleration; RAAS: Renin-angiotensin-aldosterone system.

Model 1: DNA methylation age acceleration~ age + sex + 15 medication use variables.

Model 2: DNA methylation age acceleration~ age + sex + 15 medication use variables + education + smoking + alcohol consumption + BMI + random effects.

Model 3: DNA methylation age acceleration~ age + sex + 15 medication use variables + education + smoking + alcohol consumption + BMI + random effects + white blood cell proportions.

15 Medication use variables: Diuretic + Calcium channel blockers + Beta blockers + Alpha blockers + Sympatholytics + RAAS inhibitors + statins + Sulfonylureas + Insulin + Non-narcotic analgesics + NSAID analgesics + Antidepressants + Antihistamines + Anti-anxiety medications + Narcotic analgesics.

Bold values denote significance at P < 0.05. Asterisk denotes Bonferroni corrected significance at P < 0.05/15=0.003.

Sensitivity analyses were performed to investigate whether the associations between medication use and DNAm age acceleration were independent of comorbidities. First, we repeated the multivariable models with all drug categories in the subgroup of participants with hypertension (N=779). The results from this analysis were not substantively different from the analysis in the full sample (Supplementary Table 3). The association between the use of calcium channel blockers and HannumAA was significant at the Bonferroni-corrected significance level in all models (Models 1-3). We further adjusted the multivariable models with all drug categories for the history of hypertension, stroke, coronary heart disease, and diabetes, as well as lipid levels to control for potential confounding effects (Table 3, Model 4). HannumAA was 1.27 years lower for those taking calcium channel blockers (P=0.001), and the association remained significant after further adjusting for white blood cell proportions at the Bonferroni-corrected significance level (P=0.0003 in Model 5).

**Table 3:** Association of DNA methylation age acceleration with medication use using multivariable models after further adjusting for hypertension, stroke, coronary heart disease, diabetes, and lipids.

	**HorvathAA (N=1,100)**					**HannumAA (N=1,100)**			
	**Model 4**		**Model 5**			**Model 4**		**Model 5**	
	**Beta**	**P-value**	**Beta**	**P-value**		**Beta**	**P-value**	**Beta**	**P-value**
Diuretics	0.26	0.477	0.19	0.601		0.23	0.509	0.10	0.750
Calcium channel blockers	-0.98	**0.016**	-0.98	**0.013**		-1.27	0.001*	-1.29	0.0003*
Beta blockers	0.07	0.892	-0.001	0.998		-0.98	**0.041**	-1.09	**0.015**
Alpha blockers	-1.17	0.081	-1.05	0.106		-0.13	0.833	0.07	0.899
Sympatholytics	-0.19	0.792	-0.33	0.624		-0.14	0.836	-0.40	0.513
RAAS inhibitors	0.30	0.472	0.19	0.635		-0.08	0.840	-0.22	0.547
Statins	0.13	0.855	0.13	0.852		0.19	0.783	0.25	0.692
Sulfonylureas	1.07	0.181	1.03	0.181		-0.47	0.524	-0.47	0.495
Insulins	1.02	0.209	0.89	0.254		0.49	0.515	0.13	0.852
Non-narcotic analgesics	-0.17	0.738	0.26	0.587		-0.45	0.335	0.06	0.892
NSAID analgesics	-0.93	**0.048**	-1.06	**0.020**		-0.66	0.130	-0.77	0.059
Antidepressants	0.73	0.273	0.73	0.255		0.41	0.502	0.45	0.438
Antihistamines	0.20	0.774	0.12	0.865		0.28	0.671	0.18	0.770
Antianxiety medications	0.24	0.744	0.01	0.983		0.18	0.796	0.09	0.888
Narcotic analgesics	0.30	0.743	0.07	0.938		-0.22	0.790	-0.45	0.571
	**PhenoAA (N=1,099)**					**GrimAA (N=1,099)**			
	**Model 4**		**Model 5**			**Model 4**		**Model 5**	
	**Beta**	**P-value**	**Beta**	**P-value**		**Beta**	**P-value**	**Beta**	**P-value**
Diuretics	0.59	0.248	0.65	0.167		0.16	0.560	0.27	0.282
Calcium channel blockers	-0.39	0.481	-0.28	0.586		0.25	0.394	0.34	0.231
Beta blockers	-0.24	0.741	-0.37	0.580		-0.46	0.218	-0.49	0.167
Alpha blockers	-1.84	**0.047**	-2.04	**0.018**		0.43	0.385	0.20	0.673
Sympatholytics	-0.06	0.953	-0.56	0.535		0.44	0.393	0.21	0.660
RAAS inhibitors	-0.05	0.933	-0.32	0.551		0.53	0.088	0.42	0.153
Statins	0.31	0.759	-0.33	0.730		1.12	**0.037**	0.70	0.165
Sulfonylureas	0.16	0.887	0.24	0.819		-0.19	0.747	-0.18	0.749
Insulins	0.75	0.503	0.02	0.987		0.33	0.572	0.12	0.837
Non-narcotic analgesics	-0.01	0.988	0.06	0.931		0.45	0.219	0.25	0.466
NSAID analgesics	-0.96	0.139	-1.13	0.062		0.14	0.692	0.11	0.726
Antidepressants	1.70	0.064	1.72	**0.044**		0.86	0.079	0.85	0.063
Antihistamines	-0.23	0.812	-0.10	0.911		-0.49	0.345	-0.38	0.434
Antianxiety medications	-0.90	0.383	-0.85	0.373		0.20	0.713	0.14	0.787
Narcotic analgesics	-0.36	0.770	-0.32	0.780		-0.23	0.726	-0.24	0.698

GrimAA: DNA methylations GrimAge acceleration; HannumAA: DNA methylations HannumAge acceleration; HDL: high-density lipoprotein; HorvathAA: DNA methylations HorvathAge acceleration; LDL: low-density lipoprotein; NSAID: nonsteroidal anti-inflammatory drug; PhenoAA: DNA methylations PhenoAge acceleration; RAAS: Renin-angiotensin-aldosterone system.

Model 4: DNA methylation age acceleration ~ age + sex + 15 medication use variables + education + smoking + alcohol consumption + BMI + hypertension + stroke + coronary heart disease + diabetes + HDL + Triglyceride + LDL.

Model 5: DNA methylation age acceleration ~ age + sex + 15 medication use variables + education + smoking + alcohol consumption + BMI + hypertension + stroke + coronary heart disease + diabetes + HDL + Triglyceride + LDL + Blood cell proportions.

15 Medication use variables: Diuretic + Calcium channel blockers + Beta blockers + Alpha blockers + Sympatholytics + RAAS inhibitors + statins + Sulfonylureas + Insulin + Non-narcotic analgesics + NSAID analgesics + Antidepressants + Antihistamines + Anti-anxiety medications + Narcotic analgesics.

Beta is the regression coefficient of the respective variable from the regression model as stated above.

Bold values denote significance at P < 0.05. Asterisk denotes Bonferroni corrected significance at P < 0.05/15=0.003.

### Interaction between medication use and sex on DNAm age acceleration

**Figure 1:**
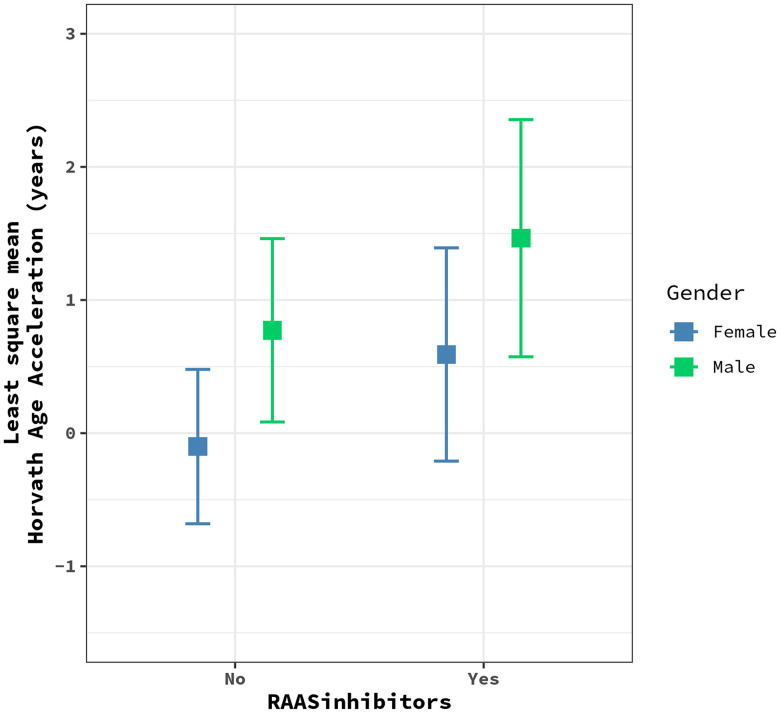
**The plot shows the effects of RAAS inhibitors on HorvathAge acceleration in males (green) vs. females (blue).** Each dot represents the least square mean of HorvathAge acceleration, and the bars represent the corresponding confidence intervals for the effect estimates. RAAS: Renin-angiotensin-aldosterone system.

We assessed whether the observed effect of medication use varied by sex by adding a medication-by-sex interaction term to the linear mixed models with one medication at a time (Supplementary Table 4). At a Bonferroni-corrected significance threshold (P=0.05/15=0.003), we observed a significant interaction between the use of RAAS inhibitors and HorvathAA after adjusting for age, sex, education, smoking, alcohol consumption, BMI, and white blood cell proportions (P=0.0015). In the sex-stratified analysis, RAAS inhibitor use was associated with 2.67-year higher HorvathAA in males after adjusting for all covariates and white blood cell proportions (P=0.001), but this association was not significant in females (Figure 1). We also included all other drug categories in the model to adjust for potential confounding effects, and the interaction remained significant (P=0.0009).

### Association between statin use and grimage components

Statin use was nominally associated with higher GrimAA in Models 1, 2, and 4. Since GrimAge is comprised of DNAm surrogates for seven proteins and smoking pack-years, we further assessed the association of statin use with each GrimAge component to identify the one(s) that may be driving the observed association. As shown in Supplementary Table 5, statin use was positively associated with DNAmADM, DNAmCystatinC, and DNAmTIMP1 after controlling for age, sex, education, smoking, alcohol consumption, and BMI at a nominal P-value threshold of 0.05. No associations were significant after Bonferroni correction for the number of GrimAge components (N=8). All nominally significant associations were attenuated after further adjusting for white blood cell proportions, suggesting that the effect of statins on GrimAge components may be partly due to changes in blood cell composition.

### Association between medication use and longitudinal change in DNAm age acceleration

We examined whether medication use influenced the change in DNAm age acceleration from Phase 1 to Phase 2 in a subset of participants whose methylation measurements were collected at both Phases (N=266) using multivariable models. We only included drug categories with N ≥ 30 at both Phases 1 and 2 (diuretics, calcium channel blockers, antihypertensives, diabetes medications, and NSAID analgesics). We evaluated the longitudinal association between medication use and change in DNAm age acceleration after categorizing medication use as “never used,” “continued,” “stopped,” and “started.”

The associations that were significant after Bonferroni correction in the longitudinal models for the change in medication use between Phase 1 and Phase 2 are shown in Table 4. The full results are presented in Supplementary Table 6. Compared to those who never used antihypertensives, those who started to take them after Phase 1 had a 0.97-year decrease in GrimAA (P=0.006 in Model 6, Table 4), and the association remained significant after further adjusting for hypertension, stroke, coronary heart disease, diabetes, and lipid levels at the Bonferroni corrected significance level (P=0.007 in Model 7). Compared to those who never used NSAID analgesics, those who started to take them after Phase 1 had an increase in HorvathAA of about 2.61 years (P=0.0005 in Model 7). Both associations remained significant with further adjustment for white blood cell proportions (Model 8).

**Table 4:** Association of NSAID analgesics and antihypertensives uses with the change rate of DNA methylation age acceleration from Phase 1 to Phase 2 using multivariable models (N=266).

			**GrimAA**			
	**Model 6**		**Model 7**		**Model 8**	
**Change in use of antihypertensives**	**Beta**	**P-value**	**Beta**	**P-value**	**Beta**	**P-value**
Never used	Ref		Ref		Ref	
Continuous use	-0.25	0.469	-0.40	0.273	-0.36	0.334
Started use after Phase 1	-0.97	0.006*	-0.97	0.007*	-0.96	0.009*
Stopped use after Phase 1	-0.47	0.394	-0.50	0.390	-0.46	0.425
			**HorvathAA**			
	**Model 6**		**Model 7**		**Model 8**	
**Change in use of NSAID analgesics**	**Beta**	**P-value**	**Beta**	**P-value**	**Beta**	**P-value**
Never used	Ref		Ref		Ref	
Continuous use	-0.12	0.917	0.10	0.933	0.12	0.920
Started use after Phase 1	2.64	0.0004*	2.61	0.0005*	2.50	0.001*
Stopped use after Phase 1	1.83	**0.017**	2.01	**0.010**	2.00	**0.011**

HannumAA: DNA methylations HannumAge acceleration; HorvathAA: DNAm HorvathAge acceleration; HDL: high-density lipoprotein; GrimAA: DNAm GrimAge acceleration; LDL: low-density lipoprotein; NSAID: nonsteroidal anti-inflammatory drug; PhenoAA: DNA methylations PhenoAge acceleration.

Only the results with any Bonferroni corrected significant association are presented here. The full results, including those for HannumAA and PhenoAA, are presented in Supplementary Table 6. Longitudinal models include subjects that had DNA methylation measured at both Phases 1 and 2. Participants whose smoking status changed between Phase 1 and phase 2 were removed.

Model 6: Change in Horvath DNA methylation age acceleration (Phase 2-Phase 1) ~ change of medication use + phase 1 covariates (age, DNAm age acceleration, sex, BMI, smoking, alcohol, education).

Model 7: Model 6 + phase 1 covariates (hypertension, stroke, coronary heart disease, diabetes, HDL, Triglyceride, LDL).

Model 8: Model 7 + blood cell proportions.

Beta is the regression coefficient of the respective variable from the regression model, as stated below.

Models were adjusted for changes in other medication use (N=5, diuretics, calcium channel blockers, antihypertensives, diabetes medications, and NSAID analgesics).

Bold values denote significance at P < 0.05. Asterisk denotes Bonferroni corrected significance at P < 0.05/5=0.01.

No other significant longitudinal effects were observed at the Bonferroni corrected significance threshold (Supplementary Table 6).

## DISCUSSION

This study investigated the cross-sectional and longitudinal associations of commonly used pharmaceuticals with four DNAm aging clocks in a large African American cohort. We found that DNAm age acceleration was associated with commonly used medications, but the strength of the associations varied by clock. Specifically, the use of calcium channel blockers was associated with 1.27-year lower HannumAA in the cross-sectional analysis even after further controlling for confounding diseases or traits. The association remained significant in a sensitivity analysis, including only participants with hypertension. In addition, RAAS inhibitor use was associated with 2.67-year higher HorvathAA only in males. In longitudinal analysis, the use of antihypertensives among those who began taking it after Phase 1 was associated with a 0.97-year decrease in GrimAA while the use of NSAID analgesics was associated with a 2.61-year increase in HorvathAA.

To our knowledge, there have been a limited number of other studies investigating the association between medication use and epigenetic aging clocks. Gao et al. [27] recently evaluated the association between antihypertensives and DNAm age acceleration in a longitudinal study of older male participants of European ancestry (N=546). They found a significant association between any antihypertensive medication use and higher HorvathAA, which is in contrast to our findings that some types of antihypertensives were associated with lower HorvathAA. In a cross-sectional analysis of the first visit with the subset of participants who had hypertension in Gao et al., calcium channel blockers were not associated with HorvathAA; however, the association in GENOA was significant at a Bonferroni corrected threshold. Potential explanations could include differences in ancestry (NAS: European ancestry; GENOA: African ancestry), mean age (NAS: 71.6 years; GENOA 57.0 years), recruitment criteria of participants (NAS: free of chronic disease; GENOA: sibships with at least 2 individuals clinically diagnosed with hypertension before age 60), and sample size (NAS: 546; GENOA: 1,100).

Antihypertensive drug use reduces the risk of developing age-related diseases caused by hypertension, such as CVD and dementia [31, 32]. This is consistent with the observed cross-sectional association between calcium channel blockers and lower HannumAA, as well as the longitudinal association between antihypertensives and lower GrimAA in the current study. Calcium channel blockers are one of many antihypertensive drugs, but are also used for other conditions such as coronary artery disease, angina, or arrhythmia. Many of the markers used for the Hannum clock are within or near genes that have functions in aging-related conditions, such as Alzheimer’s disease [17]. Specifically, two methylation markers used for the aging clock are located in the somatostatin (*SST*) gene region, which encodes somatostatin, a peptide hormone that regulates exocrine, endocrine, and nervous system function [33]. Somatostatin is highly expressed in the brain, and its actions include inhibiting the release of excitatory neurotransmitters through voltage-gated calcium channels [34, 35]. Somatostatin has been found to be reduced in the brain and cerebrospinal fluid of Alzheimer's disease patients [36]. Antihypertensive drugs have been shown to reduce the risk of dementia, and the most significant preventive efficacy of antihypertensives against dementia has been demonstrated with calcium channel blockers [37]. The precise mechanisms of how calcium channel blockers influence dementia, in addition to their antihypertensive effect, have been actively investigated. Potential mechanisms may be through calcium homeostasis in the brain [38] and/or amyloid-beta reduction [39]. Our findings may be utilized in future studies to better understand the epigenetic mechanisms of calcium channel blockers and biological cellular aging processes, especially among the elderly population with hypertension who are at high risk of dementia. However, the results should be interpreted with caution because the association was not significant in longitudinal analysis, although the longitudinal analysis had a much smaller sample size than the cross-sectional analysis.

We observed that DNAm age was accelerated by several medications. In particular, diabetes medications, including sulfonylurea and insulin, were nominally associated with all four DNAm age acceleration measures. However, these associations were all attenuated after further adjustment for age-related diseases including diabetes. Since the use of diabetes medication indicates having diabetes by definition, our findings may be the result of a proxy outcome that reflects the influence of diabetes on cellular aging; a phenomenon referred to as “confounding by indication.” The strength of the association between statin use and higher GrimAA was reduced after adjusting for lipid levels, but remained nominally significant. One possible explanation for this effect is the adverse drug reactions (ADRs) of statin use and its influence on aging. The association between statin therapy and increased risk of development of diabetes has been previously demonstrated [40, 41], and type 2 diabetes is associated with GrimAA [20]. Interestingly, a recent study provided evidence that DNAm partially mediates the effect of statins on type 2 diabetes risk [26]. In our study, statin use was nominally associated with three surrogate marker components (plasma proteins) of GrimAge: ADM, cystatin C, and TIMP-1, all of which are associated with diabetes. Specifically, ADM is a vasodilator, and its insufficiency is associated with the pathogenesis of CVD, hypertension, and diabetes [42–44]. Cystatin-C is a biomarker of kidney function, which may be altered at the preclinical and clinical stages of diabetes [45, 46]. TIMP1 has a role in promoting cell proliferation, and is associated with CVD, diabetes, and cancer [47–49]. However, the effect of statin use on these plasma proteins was attenuated after adjusting for white blood cell proportions, indicating that the association may be due to differences in white blood cell distributions.

The interaction between use of RAAS inhibitors and sex on HorvathAA was significant after Bonferroni correction. The adverse effect of RAAS inhibitors on HorvathAA was only observed in males. Angiotensin-converting enzyme inhibitors (ACEI) and angiotensin receptor type I blockers (ARB) are two major inhibitors of the RAAS that are used as the first line of antihypertensive therapy in patients with kidney disease. They reduce the overactivity of the RAAS that is associated with the development of hypertension, CVD, and kidney disease. Differences in the RAAS between men and women may be modulated by sex hormones [50, 51], and the efficacy of RAAS treatment varies between sexes [52, 53]. The aging process increases angiotensin II, the primary effector molecule of the RAAS, and may upregulate the RAAS. This suggests the need for different RAAS therapies for the elderly [54]. More studies of the relationship between the RAAS, RAAS inhibitors, and the cellular aging process may help to improve RAAS therapies for elderly patients.

In the longitudinal analysis, we found that the use of NSAID analgesics in those who started the medication after Phase 1, compared to those who never took any NSAID analgesics, was associated with increased HorvathAA after controlling for all potential confounding covariates at a strict Bonferroni corrected significance level. This finding is not consistent with the anti-inflammatory effects of NSAID analgesics that reduce symptoms of age-related diseases. Although multiple potential factors may explain the discrepancy, including limited sample size and the relatively short follow-up time in longitudinal analysis, one important explanation is the potential adverse effects of NSAIDs. These include gastrointestinal bleeding, myocardial infarction, stroke, and renal damage [55], which may lead to morbidity and mortality, especially in patients with heart disease [55, 56]. GENOA participants have a higher baseline risk of cardiovascular events due to their recruitment criteria (e.g., essential hypertension before age 60), which may worsen these adverse effects of NSAID analgesics. An important research avenue may be to investigate whether and how epigenetic mechanisms are involved in the adverse effects of NSAIDs. However, it is important to note that the sample size for longitudinal analysis was fairly limited in our study.

Several limitations of our study must be acknowledged. Although we conducted sensitivity analyses, we were not able to fully examine whether the observed associations were driven from medication use itself or relevant diseases requiring the use of specific medications. In addition, we could not adjust for other potential confounding factors that would influence the DNAm age acceleration measures, including diet, supplements, or toxicants. We also acknowledge that the sample size for the longitudinal analysis may not be large enough to detect statistically significant associations for some medications. Future studies with larger sample sizes are warranted to more accurately estimate the longitudinal effects of medication use on change in DNAm age acceleration. The follow-up duration may also be relatively short to capture the change in DNAm age acceleration by medication use. Further, since use of a specific medication was based on participants bringing their prescription container, if a participant was taking a specific medication but did not bring the container, they were classified as not taking the medication. Therefore, we recognize that some participants classified as not taking a particular medication may actually have been taking the medication. This would then bias the results toward the null. Where we detect differences between those who were classified as taking a medication versus not, the differences we detected may actually be smaller than the true differences. In addition, we collected current medication use at two exam timepoints, but had no data for the medications that participants were taking between the two exams. Finally, we cannot generalize our findings to non-hypertensive populations or populations of different races/ancestries.

A notable strength of our research includes using four epigenetic aging clocks to capture the influence of medication use on different aspects of biological aging. The strength of association with each medication varied by clock, which suggests that different clocks may capture adverse or protective effects from different medications. DNAm aging clocks, after adjusting for age, are not highly correlated, and most of the DNAm aging clocks are, in fact, largely comprised of non-overlapping CpG sites [23]. To our knowledge, this is the first study that comprehensively examined the association of commonly used medications with multiple DNAm clocks in the African ancestry population using both cross-sectional and longitudinal approaches.

In summary, our results suggest that DNAm age acceleration is associated with commonly used medications in African Americans, but that associations varied by clock. Notably, we found a significant cross-sectional association of calcium blocker use with lower HannumAA and a longitudinal association of antihypertensives with lower GrimAA after controlling for potential confounding factors, including hypertension. This study sheds light on the epigenetic effects of pharmaceuticals on aging at the cellular level captured by multiple DNA methylation aging clocks. However, further investigation is warranted to replicate our findings and confirm that the associations were driven by the medication use itself. These findings will be valuable for investigators who plan to use epigenetic biomarkers in pharmaceutical studies of age-related diseases. Future studies further investigating the influence of individual medication use on cellular aging are needed to better understand their potential beneficial and deleterious epigenetic mechanisms.

## MATERIALS AND METHODS

### Study sample

The GENOA study is a community-based longitudinal study that was initiated to identify the genetic effects of hypertension and related target organ damage [57]. In the first phase of GENOA (Phase 1: 1996-2001), European American and African American sibships with at least 2 individuals who were clinically diagnosed with hypertension before age 60 were recruited. All other siblings were invited to participate, regardless of hypertension status. Exclusion criteria included secondary hypertension, alcoholism or drug abuse, pregnancy, insulin-dependent diabetes mellitus, active malignancy, or serum creatinine levels >2.5mg/dL [57]. In Phase 1, 1,583 European Americans (Rochester, MN) and 1,854 African Americans (Jackson, MS) were enrolled. In the first follow-up phase of GENOA (Phase 2: 2000-2004), 1,239 non-Hispanic white and 1,482 African American participants were successfully followed up, and their potential target organ damage from hypertension was measured. Demographics, medical history, clinical characteristics, information on medication use, and blood samples were obtained in each phase. In GENOA, DNA methylation levels were measured only in African American participants using blood samples collected at Phases 1 and 2. African American GENOA participants with DNA methylation available at Phase 1 were included in the current cross-sectional analysis (N=1,100). In the longitudinal analysis, those with available DNA methylation measurements at both Phases 1 and 2 and without changes in smoking status between phases were included (N=266). Written informed consent was obtained from all subjects, and approval was granted by participating institutional review boards (University of Michigan, University of Mississippi Medical Center, and Mayo Clinic).

### Methylations measures

A total of 1,106 samples at Phase 1 and 304 samples at Phase 2 were assessed using the Illumina HumanMethylationEPIC BeadChip. First, raw IDAT files were imported using the Minfi R package [58]. We used the shinyMethyl R package [59] to visualize the raw intensity data and identify sex mismatches and outliers, which were removed. We also obtained the detection p-value for each sample at each probe, and individual probes with detection p-value <10^-16^ were considered to be successfully detected [60]. Samples and probes with the detection rate <10% were removed [60]. Samples with incomplete bisulfite conversion identified using the QCinfo() function in the ENmix R package were removed [61]. We also checked sample identity using the 59 SNP probes implemented in the EPIC chip and removed mismatched samples. Next, Noob was used for individual background and dye-bias normalization [62]. Since two types of probes are present on the EPIC BeadChip (Infinium I and Infinium II), we used the Regression on Correlated Probes (RCP) method to adjust for probe-type bias [63]. ComBat, an empirical Bayes batch-correction method, was used sequentially to remove those batch effects (Johnson, 2007). We performed principal variance component analysis (PVCA) to quantify the variance explained by the known batch variables before and after batch adjustment to ensure that no single batch factors explained more than 3% of the variance. After exclusions, a total of 857,121 probes in 1,100 samples at Phase 1 and 294 samples at Phase 2 remained for analysis.

### DNAm age calculation and blood cell counts

To calculate HorvathAge, methylation beta values were uploaded to the online Horvath epigenetic age calculator [18]. HorvathAge was estimated based on 353 CpGs [18]. This epigenetic clock captures chronological age across multiple tissue types and in different age groups. HorvathAA is the residual from regressing HorvathAge on chronological age. HannumAge was estimated based on 71 CpG sites using a single tissue (whole blood) [17], which captures chronological age. HannumAge acceleration (HannumAA) is the residual from regressing HannumAge on chronological age. PhenoAge was estimated using 513 CpG sites, and the PhenoAA measure is the residual from regressing PhenoAge on chronological age [19]. PhenoAge captures the effect of phenotypic aging as it was trained on many aging-related clinical measurements, including albumin, creatinine, glucose, C-reactive protein, alkaline phosphatase, white blood cell count, chronological age, and other metrics. GrimAge was estimated using 1,030 CpG sites, and GrimAA is the residual from regressing GrimAge on chronological age [20]. GrimAge captures the effects of the number of cigarettes smoked in pack-years as well as seven plasma proteins associated with mortality, including adrenomedullin, beta-2 microglobulin, growth differentiation factor 15, Cystatin C, leptin, plasminogen activation inhibitor 1, and tissue inhibitor metalloproteinase 1 [20]. Since GrimAge is a composite measure, we wanted to better understand the effects of pharmaceuticals on each component of GrimAge, so we additionally obtained the DNAm surrogates of all eight individual items from the online calculator. It is important to note that the EPIC array is missing 19 sites used to construct HorvathAge, and six sites that are used to calculate HannumAge; however, previous studies have demonstrated that this does not impact the performance of DNAm age predictors and acceleration measurements [64, 65]. All analyses were repeated with additional adjustment for white blood cell proportions, including CD8+ T, CD4+ T, natural killer, B cells, and granulocytes. These blood cell counts were estimated using Houseman’s method as implemented in the online epigenetic age calculator [66]. For each measure of DNAm age acceleration, outliers greater or less than 5 standard deviations from the mean were removed.

### Drug classifications and other covariates

Prescription information for medications used in the past month was collected from the labels on prescriptions provided by the participants at each GENOA visit. The Medi-Span Therapeutic Classification (MTC) was utilized to codify the pharmacological class of each prescription using the first six digits. For the current analysis, we categorized medications into major classes using the first two digits of the MTC code. Major drug categories with N < 30 at Phase 1 were excluded from the analysis. The 12 major medication categories included diuretics, calcium channel blockers, beta blockers, antihypertensives, antihyperlipidemic, diabetes medication, non-narcotic analgesics, NSAID analgesics, antidepressants, antihistamines, antianxiety medication, and narcotic analgesics. Categories of antihypertensive, antihyperlipidemic, and diabetes medications were further subcategorized using the third digit of the MTC codes and were included in the analysis if N ≥ 30 at Phase 1: antihypertensives were further subcategorized into alpha blockers, sympatholytic, and RAAS inhibitors; antihyperlipidemics included statins as a sub-category; and diabetes medications were further classified into sulfonylurea and insulin. Although diuretics, calcium channel blockers, and beta blockers are often prescribed as antihypertensive medications, they have different MTC codes because they are used for other conditions as well, such as congestive heart failure, angina, and arrhythmia. As a result, a total of 15 drug categories were available at Phase 1 for the cross-sectional analysis. We only included major drug categories with N ≥ 30 at both Phases 1 and 2 (diuretics, calcium channel blockers, antihypertensives, diabetes medications, and NSAID analgesics) for the longitudinal analysis due to the limited sample size.

Self-reported smoking status was obtained at Phases 1 and 2 of GENOA. Current smokers were defined as individuals who reported smoking at the time of the exam and had smoked at least 100 cigarettes in one’s lifetime. Former smokers were individuals who had smoked at least 100 cigarettes in one’s lifetime but do not currently smoke. Never smokers were individuals who had not smoked more than 100 cigarettes in one’s lifetime. Educational attainment was collected at GENOA Phase 1 as the self-reported highest degree, as well as years of education. Participants were grouped as having less than a high school degree (<12 years of education), high school degree or equivalent (12 years or GED), and some college and above (> 12 years). Alcohol consumption was determined based on the number of drinks per week. Because the distribution of alcohol consumption was skewed, it was log-transformed prior to the analysis. Weight and height were collected at both phases, and BMI was calculated. Participants’ medical conditions were self-reported and confirmed during the medical exam. Participants were classified as having hypertension if they were taking antihypertensive medications, had a systolic blood pressure ≥ 140 mmHg, or had a diastolic blood pressure ≥ 90 mmHg. Patients taking diabetes medication or with glucose level measurement ≥ 126mg/dL were categorized as having diabetes.

### Statistical analyses

We used Chi-square tests to assess independence among medication categories. We then performed cross-sectional analysis to assess the associations between medication use and the four DNAm age acceleration measures at Phase 1. For each DNAm age acceleration measure, multivariable linear mixed models were constructed with all of the drug categories as the predictors and DNAm age acceleration as the outcome, adjusting for age and sex, with batch effects and familial relationships as random effects (Model 1). We included all of the drug categories in a single model to capture the unique contribution of each medication. We also included socioeconomic and lifestyle factors that have been previously associated with DNAm age acceleration, including education, smoking status, alcohol consumption, and BMI (Model 2). Since the DNAm age clocks are correlated with white blood cell counts, we additionally adjusted for white blood cell proportions to assess whether any of the associations were due to differences in blood cell composition (Model 3). For the three major drug categories (antihypertensives, antihyperlipidemics, and diabetes medications) that had sub-categories, we included only the sub-categories into the multivariable model. Since we evaluated multiple medications, Bonferroni correction was used to account for multiple comparisons. However, the measures of medication use are not independent, and the Bonferroni approach is conservative in this setting, so we also considered a nominal significance level (P<0.05).

Since almost everyone treated with a drug has, by definition, the relevant disease to begin with, it is important to assess whether the associations of medication use with DNAm age acceleration were independent of relevant participants’ disease status or traits. To address this issue, we ran two sensitivity analyses, as follows. First, we repeated the analyses with multivariable models among a subset of participants who had hypertension since GENOA is a predominately hypertensive cohort. Second, we repeated the analyses in all participants controlling for age-related diseases and lipid levels (hypertension, stroke, coronary heart disease, diabetes, high-density lipoproteins (HDL), triglycerides, and low-density lipoproteins (LDL) (Model 4). Model 5 additionally adjusted for white blood cell proportions.

We assessed whether the observed effects of medication use on DNAm age acceleration differed by gender. For medication-by-sex interactions significant after Bonferroni correction (P<0.05/15 = 0.003), we further examined the sex-specific effects of the medication on DNAm age acceleration. Since GrimAge was comprised of DNAm surrogates of eight individual items (ADM, B2M, GDF15, CystatinC, Leptin, PAI1, TIMP1, and PACKYRS), we also assessed the observed effect with each component to identify those that may drive the association for any medication categories that were associated with GrimAA in Model 4.

Last, we conducted longitudinal analysis for drug categories with N ≥ 30 at both Phase 1 and Phase 2 on a subset of samples (N=266) whose DNA methylation was measured at both Phases. Batch effects and familial relationships were adjusted as random effects. Because smoking status substantially influences DNA methylation, we removed 27 participants whose smoking status changed between Phases. We considered the change in medication use between Phases by setting the change in DNAm age acceleration between Phases as the outcome and the change in medication use as the predictor. The change in medication use was categorized as "Never used" (reference category), "Continued," "Stopped," and "Started." Model 6 covariates included Phase 1 age, sex, education, BMI, smoking status, alcohol consumption, and DNAm age acceleration, and Model 7 additionally controlled for Phase 1 hypertension, stroke, coronary heart disease, diabetes, HDL, triglycerides, and LDL. We repeated the analyses with further adjustment for white blood cell counts to assess whether the association observed was independent of the changes in blood cell composition (Model 8).

## Supplementary Material

Supplementary Figures

Supplementary Tables
