# Supplementary material for: Accelerated DNA methylation age and medication use among African Americans
Source: Aging (Albany NY). 2021 Jun 3;13(11):14604–29. doi: 10.18632/aging.203115 (PMC8221348; doi:10.18632/aging.203115)
Supplement: Supplementary Figures [file aging-13-203115-s001.pdf]

SUPPLEMENTARY FIGURES

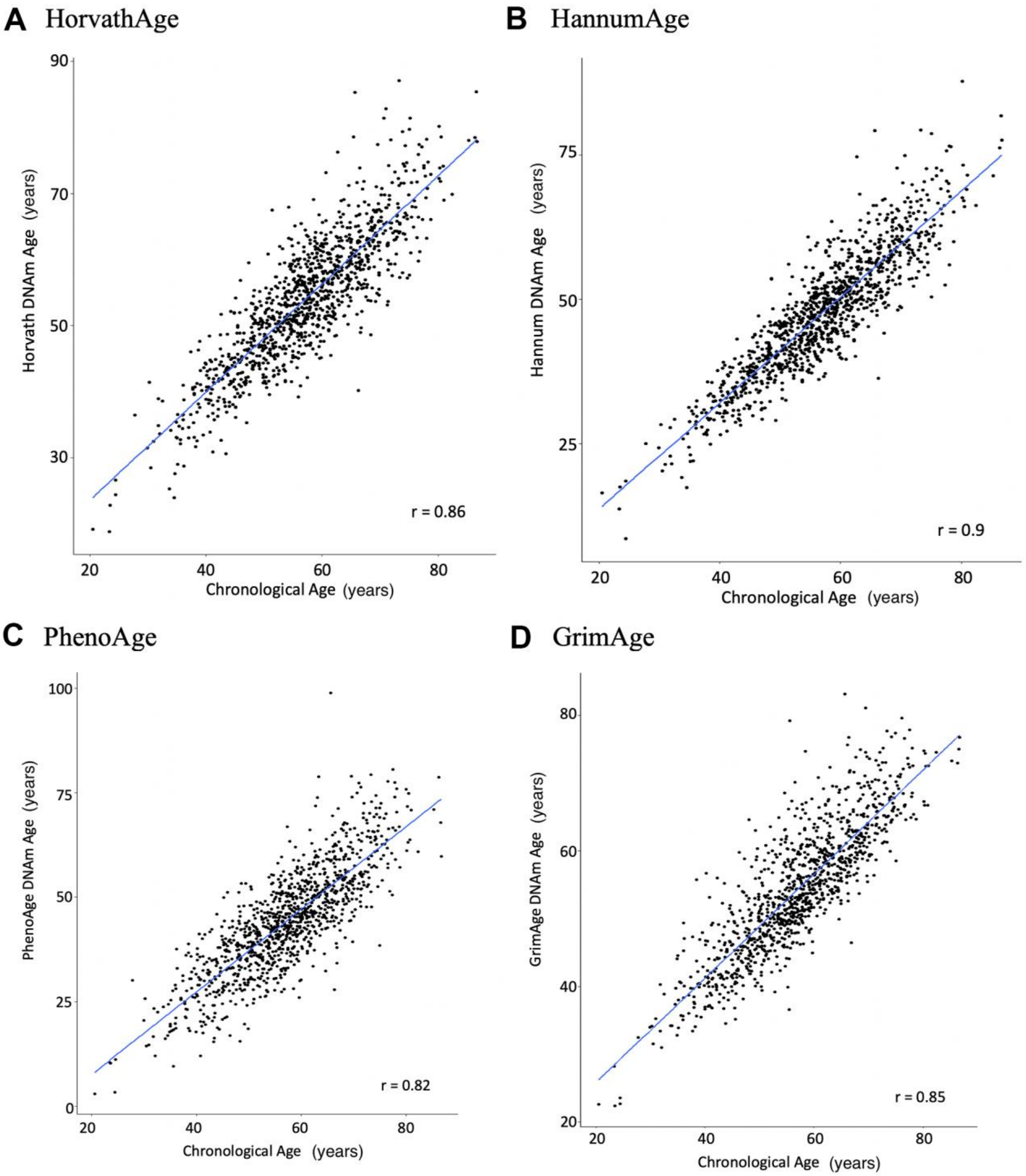

Supplementary Figure 1. Correlation between chronological age and DNAm age estimated by four epigenetic clocks. Scatterplots with Pearson correlation coefficients between chronological age and DNA methylation age estimated by the HorvathAge (**A**), HannumAge (**B**), PhenoAge (**C**), and GrimAge (**D**) clocks. DNAm: DNA methylation; r: Pearson correlation coefficient.

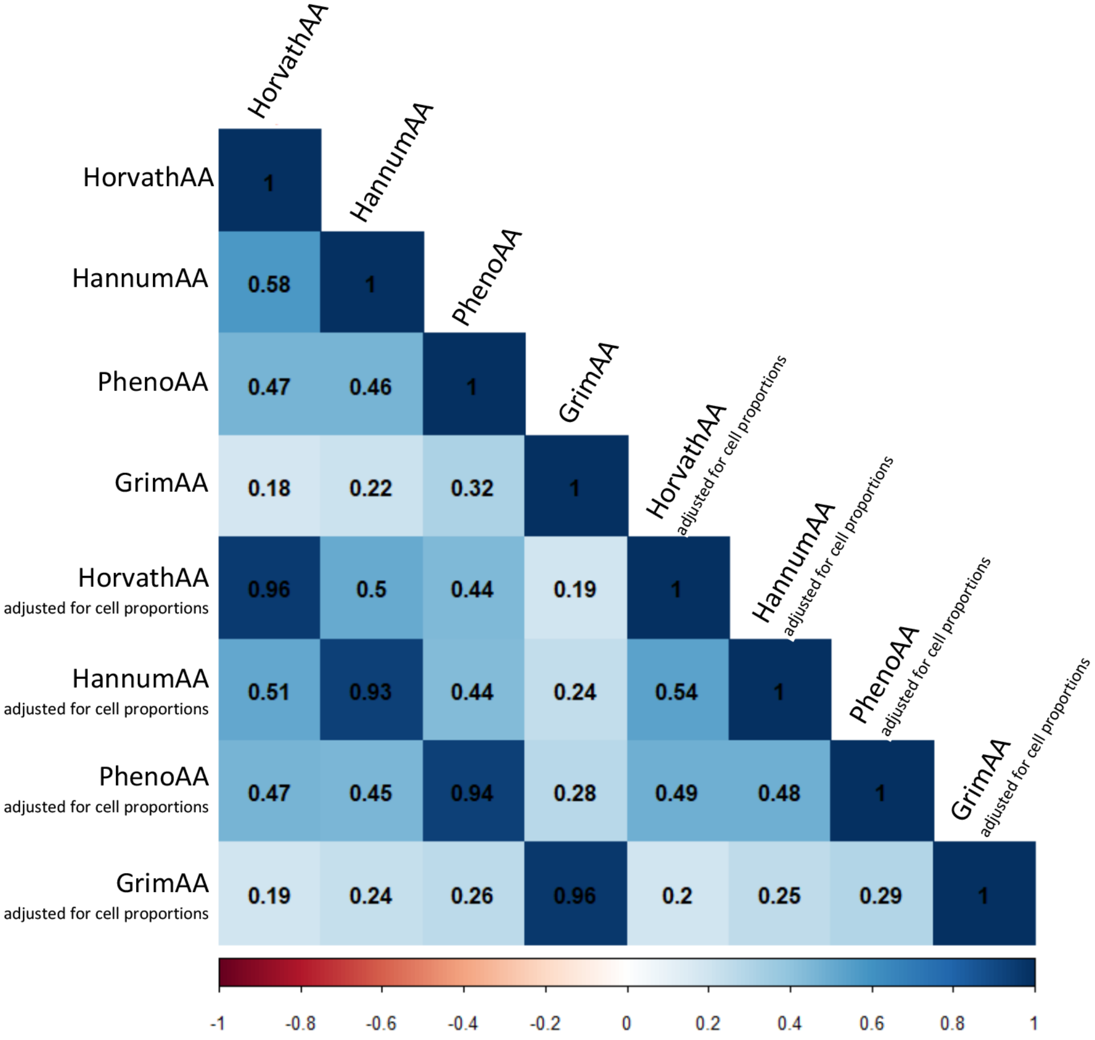

Supplementary Figure 2. Pearson correlation coefficients for DNA methylation age acceleration metrics estimated by four epigenetic clocks.
