# Supplementary material for: Accelerated DNA methylation age and medication use among African Americans
Source: Aging (Albany NY). 2021 Jun 3;13(11):14604–29. doi: 10.18632/aging.203115 (PMC8221348; doi:10.18632/aging.203115)
Supplement: Supplementary Tables [file aging-13-203115-s002.pdf]

SUPPLEMENTARY TABLES

Supplementary Table 1. Medication use by first two digits of medi-span therapeutic classification (MTC) drug classification code in the genetic epidemiology network of arteriopathy (GENOA).

		Cross-Sectional (N=1,100)	Longitudinal (N=266)	
	**First two digitsof MTC code**	**Phase 1**	**Phase 1**	**Phase 2**
Diuretics	D37	377	79	109
Calcium channel blockers	D34	221	58	77
Beta blockers	D33	118		
Antihypertensives	D36	321	73	107
Alpha blockers		59		
Sympatholytics		54		
RAAS inhibitors		207		
Antihyperlipidemics	D39	62		
Statins		51		
Diabetes medications	D27	164	32	56
Sulfonylureas		82		
Insulins		78		
Non-narcotic analgesics	D64	141		
NSAID analgesics	D66	130	36	38
Antidepressants	D58	60		
Antihistamines	D41	53		
Antianxiety medications	D57	48		
Narcotic analgesics	D30	30		

NSAID: nonsteroidal anti-inflammatory drug; RAAS: renin–angiotensin–aldosterone system.
Number of participants taking each drug category is displayed. Medication use in GENOA was categorized using the first two digits of MTC drug classification code. For categories of antihypertensives, antihyperlipidemics, and diabetes medication, sub-categories were included. Drug categories with N < 30 were excluded.

Supplementary Table 2. P-value from Chi-square test of independence among medication categories.

	Calcium channel blockers	Beta blockers	Alpha blockers	Sympatholytics	RAAS inhibitors	Statins	Sulfonylureas	Insulins	Non-narcotic analgesics	NSAID analgesics	Antidepressants	Antihistamines	Antianxiety medications	Narcotic analgesics
Diuretics	3.1E-04	4.7E-11	0.02	4.2E-04	1.3E-10	2.5E-03	0.02	0.02	2.7E-04	8.7E-05	0.10	5.6E-03	0.03	0.28
Calcium channel blockers		1.00	0.91	0.90	0.69	0.01	0.02	0.16	9.6E-10	0.75	3.7E-05	0.09	0.29	0.48
Beta blockers			0.35	0.14	0.94	0.06	1.00	0.42	2.8E-05	0.89	0.03	0.20	8.2E-06	0.30
Alpha blockers				1.00	0.89	1.00	0.11	0.87	0.24	0.83	0.18	0.68	0.06	1.00
Sympatholytics					0.81	1.00	0.80	0.72	0.86	0.63	0.78	0.95	1.00	0.38
RAAS inhibitors						0.15	9.1E-11	5.4E-11	4.1E-06	0.82	0.68	0.36	0.04	1.00
Statins							0.35	0.01	0.03	0.51	0.65	0.49	0.11	1.00
Sulfonylureas								0.89	0.02	1.00	0.31	0.06	1.00	0.60
Insulins									3.5E-07	0.23	0.03	0.89	0.53	1.00
Non-narcotic analgesics										0.03	1.8E-05	0.47	0.55	0.46
NSAID analgesics											0.32	3.3E-04	1.8E-03	6.4E-04
Antidepressants												4.3E-03	0.57	1.00
Antihistamines													0.03	0.96
Antianxiety medications														1.00

NSAID: nonsteroidal anti-inflammatory drug; RAAS: Renin-angiotensin-aldosterone system.
P < 0.05 is highlighted.

Supplementary Table 3. Association of DNA methylation age acceleration with medication use using multivariable models among those with hypertension.

	HorvathAA (N=779)						HannumAA (N=779)					
	Model 1		Model 2		Model 3		Model 1		Model 2		Model 3	
	Beta	P-value	Beta	P-value	Beta	P-value	Beta	P-value	Beta	P-value	Beta	P-value
Diuretics	0.16	0.680	0.24	0.536	0.17	0.652	0.27	0.455	0.34	0.350	0.25	0.460
Calcium channel blockers	-1.21	**0.005**	-1.17	**0.006**	-1.13	**0.006**	-1.30	**0.001***	-1.36	**0.001***	-1.33	**0.0003***
Beta blockers	-0.13	0.815	-0.06	0.907	-0.15	0.775	-1.16	**0.022**	-1.04	**0.039**	-1.13	**0.016**
Alpha blockers	-1.37	0.053	-1.16	0.101	-1.04	0.128	-0.34	0.603	-0.22	0.738	0.00	0.997
Sympatholytics	-0.21	0.775	-0.34	0.642	-0.55	0.438	0.11	0.876	-0.13	0.844	-0.40	0.532
RAAS inhibitors	0.17	0.705	0.05	0.911	-0.04	0.923	-0.10	0.817	-0.16	0.705	-0.27	0.489
Statins	-0.01	0.988	0.11	0.884	0.15	0.844	0.15	0.839	0.30	0.680	0.40	0.561
Sulfonylureas	1.39	**0.034**	1.29	**0.049**	1.32	**0.038**	0.64	0.285	0.49	0.422	0.56	0.329
Insulins	1.37	**0.041**	1.17	0.081	1.05	0.103	1.46	**0.018**	1.42	**0.022**	1.16	**0.047**
Non-narcotic analgesics	-0.04	0.934	-0.31	0.566	0.15	0.781	-0.48	0.324	-0.60	0.224	-0.09	0.852
NSAID analgesics	-0.68	0.226	-0.75	0.180	-0.85	0.116	-0.48	0.351	-0.48	0.353	-0.55	0.250
Antidepressants	0.75	0.328	0.70	0.354	0.66	0.365	0.49	0.488	0.41	0.553	0.38	0.567
Antihistamines	0.10	0.904	0.06	0.937	-0.04	0.961	0.28	0.710	0.31	0.682	0.22	0.756
Antianxiety medications	-0.14	0.873	-0.12	0.888	-0.37	0.653	-0.17	0.829	-0.15	0.851	-0.23	0.755
Narcotic analgesics	-0.25	0.840	-0.24	0.846	-0.36	0.763	-0.01	0.990	-0.29	0.801	-0.45	0.672
	PhenoAA (N=778)						GrimAA (N=779)					
	Model 1		Model 2		Model 3		Model 1		Model 2		Model 3	
	Beta	P-value	Beta	P-value	Beta	P-value	Beta	P-value	Beta	P-value	Beta	P-value
Diuretics	0.63	0.246	0.72	0.187	0.79	0.119	-0.25	0.469	0.13	0.644	0.24	0.373
Calcium channel blockers	-0.40	0.505	-0.50	0.397	-0.44	0.414	0.38	0.310	0.27	0.389	0.30	0.302
Beta blockers	-0.11	0.881	-0.03	0.973	-0.27	0.693	-0.39	0.413	-0.23	0.552	-0.36	0.333
Alpha blockers	-2.18	**0.027**	-2.06	**0.036**	-2.25	**0.013**	0.39	0.534	0.42	0.410	0.17	0.731
Sympatholytics	-0.08	0.941	-0.30	0.768	-0.72	0.445	0.68	0.294	0.44	0.405	0.20	0.690
RAAS inhibitors	0.05	0.939	0.03	0.955	-0.27	0.638	0.45	0.254	0.63	0.050	0.48	0.114
Statins	0.33	0.762	0.35	0.745	-0.30	0.766	1.89	**0.006**	1.28	**0.024**	0.83	0.122
Sulfonylureas	1.81	**0.048**	1.77	0.053	1.80	**0.033**	0.77	0.177	0.74	0.118	0.60	0.179
Insulins	2.17	**0.020**	2.15	**0.020**	1.41	0.099	0.60	0.299	1.07	**0.026**	0.80	0.082
Non-narcotic analgesics	0.96	0.194	0.77	0.295	0.74	0.285	0.87	0.062	0.78	**0.043**	0.49	0.179
NSAID analgesics	-1.35	0.082	-1.29	0.095	-1.24	0.079	-0.40	0.412	-0.10	0.797	-0.07	0.848
Antidepressants	1.61	0.127	1.48	0.158	1.41	0.144	1.03	0.121	0.71	0.194	0.75	0.147
Antihistamines	0.13	0.908	0.34	0.766	0.62	0.555	-0.94	0.189	-0.39	0.507	-0.26	0.634
Antianxiety medications	-1.67	0.165	-1.54	0.198	-1.58	0.154	0.52	0.494	0.22	0.723	0.09	0.878
Narcotic analgesics	0.55	0.744	-0.11	0.950	-0.52	0.736	1.03	0.341	-0.28	0.750	-0.61	0.470

GrimAA: DNA methylation GrimAge acceleration; HannumAA: DNA methylations HannumAge acceleration; HorvathAA: DNA methylations HorvathAge acceleration; NSAID: nonsteroidal anti-inflammatory drug; PhenoAA: DNA methylations PhenoAge acceleration; RAAS: Renin-angiotensin-aldosterone system.

Model 1: DNA methylation age acceleration~ age + sex + 15 medication use variables.

Model 2: DNA methylation age acceleration~ age + sex + 15 medication use variables + education + smoking + alcohol consumption + BMI + random effects.

Model 3: DNA methylation age acceleration~ age + sex + 15 medication use variables + education + smoking + alcohol consumption + BMI + random effects + white blood cell proportion.

Medication use variables: Diuretic + Calcium channel blockers + Beta blockers + Alpha blockers + Sympatholytics + RAAS inhibitors + Statins + Sulfonylureas + Insulin + Non-narcotic analgesics + NSAID analgesics + Antidepressants + Antihistamines + Anti-anxiety medications + Narcotic analgesics.

Bold values denote significance at $P < 0.05$. Asterisk denotes Bonferroni corrected significance at $P < 0.05/15=0.003$.

Supplementary Table 4. Interactions between medication use and sex on DNAm age acceleration.

	HorvathAA (N=1,100)						HannumAA (N=1,100)					
	Model 1		Model 2		Model 3		Model 1		Model 2		Model 3	
	Beta	P-value	Beta	P-value	Beta	P-value	Beta	P-value	Beta	P-value	Beta	P-value
Diuretics	-0.83	0.276	-0.63	0.402	-0.19	0.790	-0.43	0.543	-0.36	0.608	0.16	0.813
Calcium channel blockers	-0.28	0.740	-0.34	0.682	0.00	0.998	-0.03	0.965	-0.09	0.912	0.09	0.897
Beta blockers	-0.83	0.463	-0.75	0.506	-0.93	0.394	1.60	0.125	1.67	0.110	1.36	0.165
Alpha blockers	-1.09	0.415	-1.11	0.404	-1.70	0.186	-0.91	0.462	-1.10	0.374	-1.87	0.107
Sympatholytics	1.50	0.314	1.78	0.228	1.48	0.300	0.33	0.813	0.52	0.707	0.21	0.868
RAAS inhibitors	-2.34	**0.004**	-2.27	**0.006**	-2.51	**0.0015***	-1.04	0.173	-1.00	0.187	-1.44	**0.044**
Statins	1.54	0.346	1.92	0.239	2.36	0.133	0.23	0.882	0.36	0.810	0.84	0.555
Sulfonylureas	-1.57	0.192	-1.45	0.227	-1.39	0.230	0.03	0.982	-0.15	0.894	-0.34	0.748
Insulins	-2.55	**0.048**	-2.22	0.085	-2.45	**0.049**	-0.60	0.620	-0.37	0.756	-0.67	0.553
Non-narcotic analgesics	-0.01	0.995	0.07	0.942	-0.06	0.948	0.80	0.360	0.72	0.407	0.51	0.532
NSAID analgesics	0.30	0.767	0.19	0.848	1.09	0.262	1.00	0.288	0.90	0.336	1.77	**0.043**
Antidepressants	-0.26	0.868	-0.17	0.916	-0.80	0.594	1.01	0.488	1.15	0.425	0.26	0.846
Antihistamines	0.31	0.851	0.27	0.870	0.20	0.902	-0.18	0.905	-0.37	0.806	-0.52	0.713
Antianxiety medications	0.01	0.997	0.34	0.831	0.43	0.782	2.57	0.084	2.75	0.065	2.13	0.130
Narcotic analgesics	3.00	0.107	2.69	0.148	2.39	0.182	0.69	0.690	0.61	0.721	0.40	0.803
	**PhenoAA (N=1,099)**						**GrimAA (N=1,099)**					
	**Model 1**		**Model 2**		**Model 3**		**Model 1**		**Model 2**		**Model 3**	
	**Beta**	**P-value**	**Beta**	**P-value**	**Beta**	**P-value**	**Beta**	**P-value**	**Beta**	**P-value**	**Beta**	**P-value**
Diuretics	0.09	0.928	0.17	0.872	0.44	0.652	0.31	0.661	0.35	0.536	0.33	0.532
Calcium channel blockers	-1.25	0.276	-1.34	0.242	-0.65	0.544	-0.94	0.219	-0.95	0.126	-0.66	0.257
Beta blockers	1.30	0.400	0.91	0.557	0.85	0.556	0.91	0.380	-0.01	0.992	0.21	0.787
Alpha blockers	0.94	0.608	0.66	0.717	-0.47	0.781	0.69	0.574	0.45	0.652	0.26	0.780
Sympatholytics	-0.54	0.793	-0.15	0.941	-0.93	0.626	-0.63	0.644	0.02	0.985	-0.45	0.659
RAAS inhibitors	-0.95	0.402	-0.81	0.472	-1.23	0.242	-1.01	0.184	-0.59	0.330	-0.60	0.291
Statins	-2.51	0.267	-1.97	0.380	-1.26	0.548	-1.00	0.501	-0.52	0.663	-0.34	0.761
Sulfonylureas	-0.13	0.937	-0.31	0.855	0.45	0.772	-0.20	0.857	-0.66	0.459	-0.10	0.905
Insulins	0.00	0.999	0.30	0.866	-0.47	0.778	0.40	0.739	0.42	0.661	0.03	0.976
Non-narcotic analgesics	0.08	0.951	0.11	0.934	0.32	0.792	-0.75	0.385	-0.98	0.157	-0.79	0.227
NSAID analgesics	0.62	0.656	0.50	0.719	0.94	0.467	1.59	0.086	1.32	0.077	1.20	0.088
Antidepressants	1.52	0.480	1.55	0.466	1.23	0.534	-0.13	0.929	0.18	0.874	0.30	0.783
Antihistamines	1.02	0.653	1.04	0.645	0.15	0.942	2.68	0.076	2.46	**0.043**	2.02	0.077
Antianxiety medications	0.08	0.971	-0.38	0.865	-1.41	0.505	0.49	0.740	-1.28	0.285	-1.35	0.231
Narcotic analgesics	1.96	0.441	1.76	0.488	0.93	0.693	-0.11	0.948	-0.50	0.716	-0.90	0.485

GrimAA: DNA methylation GrimAge acceleration; HannumAA: DNA methylations HannumAge acceleration; HorvathAA: DNA methylations HorvathAge acceleration; NSAID: nonsteroidal anti-inflammatory drug; PhenoAA: DNA methylations PhenoAge acceleration; RAAS: Renin-angiotensin-aldosterone system.

Model 1: DNA methylation age acceleration ~ age + sex + medication use (one at a time) + medication use*sex.

Model 2: DNA methylation age acceleration ~ age + sex + medication use (one at a time) + education + smoking + alcohol consumption + BMI + medication use*sex.

Model 3: DNA methylation age acceleration ~ age + sex + medication use (one at a time) + education + smoking + alcohol consumption + BMI + white blood cell proportions + medication use*sex.

Beta is the regression coefficient of the interaction term between respective medication variable and sex from the regression model as stated above.

Bold values denote significance at $P < 0.05$.

Asterisk denotes Bonferroni corrected significance at $P < 0.05/15=0.003$.

Supplementary Table 5. Associations of GrimAge components with statin use using multivariable models (N=1,100).

GrimAge Component	Model 1			Model 2			Model 3		
	Beta	SD	P-value	Beta	SD	P-value	Beta	SD	P-value
DNAmADM	4.91	2.36	**0.038**	5.16	2.35	**0.028**	3.35	2.30	0.145
DNAmB2M	8345.00	20252.93	0.680	8116.31	20282.21	0.689	6713.34	20211.28	0.740
DNAmCystatinC	7434.12	3236.05	**0.022**	7884.45	3239.36	**0.015**	4816.55	3007.28	0.110
DNAmGDF15	25.80	15.88	0.105	21.82	15.55	0.161	12.42	15.34	0.418
DNAmLeptin	-59.50	258.21	0.818	-8.68	257.52	0.973	53.35	258.07	0.836
DNAmPACKYRS	3.04	1.82	0.094	1.11	1.22	0.366	0.68	1.22	0.579
DNAmPAI1	596.35	365.89	0.103	622.38	347.12	0.073	449.63	343.95	0.191
DNAmTIMP1	334.04	143.42	**0.020**	362.00	143.71	**0.012**	232.06	136.51	0.089

DNAmADM: DNAm surrogate of adrenomedullin (ADM); DNAmB2M: DNAm surrogate of beta-2 microglobulin (B2M), DNAmGDF15: DNAm.
surrogate of growth differentiation factor 15 (GDF15); DNAmCystatinC: DNAm surrogate of Cystatin C (CystatinC); DNAmLeptin: DNAm.
surrogate of leptin (Leption); DNAmPAI1: DNAm surrogate of plasminogen activation inhibitor 1 (PAI1); DNAmTIMP1: DNAm surrogate of
Issue inhibitor metalloproteinase 1 (TIMP1); DNAmPACKYRS: DNAm surrogate of the amount of cigarettes smoked (PACKYRS).
Model 1: DNA methylation GrimAge components ~ age + sex + medication use (one at a time).
Model 2: DNA methylation GrimAge components ~ age + sex + medication use (one at a time) + education + smoking + alcohol consumption + BMI.
Model 3: DNA methylation GrimAge components ~ age + sex + medication use (one at a time) + education + smoking + alcohol consumption + BMI + white blood cell proportions.
Beta is the regression coefficient of the respective variable from the regression model as stated above.
Bold values denote statistical significance at P < 0.05.

Supplementary Table 6. Association of medication use with change rate of DNA methylation age acceleration from Phase 1 to Phase 2 using multivariable models (N=266).

	Change of medication use	HorvathAA						HannumAA					
		Model 1		Model 2		Model 3		Model 1		Model 2		Model 3	
		Beta	P-value	Beta	P-value	Beta	P-value	Beta	P-value	Beta	P-value	Beta	P-value
Diuretics	Never used	Ref		Ref		Ref		Ref		Ref		Ref	
	Continuous use	0.98	0.112	1.27	0.054	1.25	0.063	0.10	0.834	-0.14	0.787	-0.21	0.687
	Started use after Phase 1	-0.74	0.246	-0.59	0.370	-0.52	0.434	0.16	0.762	0.06	0.915	0.04	0.934
	Stopped use after Phase 1	0.56	0.540	0.67	0.502	0.69	0.509	0.38	0.608	0.10	0.902	0.06	0.940
Calcium channel blockers	Never used	Ref		Ref		Ref		Ref		Ref		Ref	
	Continuous use	0.59	0.331	0.80	0.215	0.84	0.208	0.13	0.788	0.02	0.976	-0.03	0.950
	Started use after Phase 1	-0.56	0.414	-0.39	0.579	-0.25	0.729	0.66	0.231	0.61	0.271	0.57	0.315
	Stopped use after Phase 1	0.95	0.372	1.28	0.248	1.28	0.261	0.81	0.342	0.48	0.579	0.47	0.600
Antihypertensives	Never used	Ref		Ref		Ref		Ref		Ref		Ref	
	Continuous use	-0.96	0.112	-0.71	0.278	-0.69	0.298	0.24	0.627	0.14	0.779	0.26	0.624
	Started use after Phase 1	-0.65	0.299	-0.54	0.397	-0.60	0.358	-0.52	0.300	-0.54	0.284	-0.46	0.374
	Stopped use after Phase 1	-0.34	0.727	0.09	0.932	0.10	0.922	-0.71	0.374	-1.06	0.198	-0.92	0.268
Diabetes medications	Never used	Ref		Ref		Ref		Ref		Ref		Ref	
	Continuous use	0.45	0.531	0.67	0.627	0.82	0.560	0.29	0.617	1.66	0.128	1.62	0.141
	Started use after Phase 1	0.49	0.511	0.82	0.353	0.97	0.285	-1.00	0.096	-0.30	0.662	-0.24	0.733
	Stopped use after Phase 1	-5.28	0.131	-5.42	0.195	-5.60	0.188	1.31	0.640	1.19	0.718	1.23	0.712
NSAID analgesics	Never used	Ref		Ref		Ref		Ref		Ref		Ref	
	Continuous use	-0.12	0.917	0.10	0.933	0.12	0.920	0.18	0.846	-0.25	0.783	-0.20	0.825
	Started use after Phase 1	2.64	**0.0004***	2.61	**0.001***	2.50	**0.001***	1.28	**0.032**	1.29	**0.028**	1.30	**0.030**
	Stopped use after Phase 1	1.83	**0.017**	2.01	**0.010**	2.00	**0.011**	0.19	0.760	0.41	0.497	0.43	0.485

	Change of medication use	PhenoAA						GrimAA					
		Model 1		Model 2		Model 3		Model 1		Model 2		Model 3	
		Beta	P-value	Beta	P-value	Beta	P-value	Beta	P-value	Beta	P-value	Beta	P-value
Diuretics	Never used	Ref		Ref		Ref		Ref		Ref		Ref	
	Continuous use	0.28	0.696	-0.35	0.648	-0.67	0.378	0.14	0.683	0.11	0.762	0.03	0.942
	Started use after Phase 1	0.27	0.718	0.02	0.978	0.02	0.975	-0.01	0.977	-0.08	0.838	-0.10	0.790
	Stopped use after Phase 1	-1.12	0.300	-1.65	0.158	-1.89	0.108	0.21	0.688	0.19	0.729	0.08	0.885
Calcium channel blockers	Never used	Ref		Ref		Ref		Ref		Ref		Ref	
	Continuous use	0.13	0.853	-0.34	0.646	-0.32	0.666	0.01	0.967	-0.02	0.957	-0.04	0.913
	Started use after Phase 1	-1.10	0.168	-1.37	0.093	-1.40	0.089	0.09	0.814	0.05	0.901	-0.09	0.814
	Stopped use after Phase 1	1.45	0.240	0.89	0.487	0.37	0.771	1.17	**0.049**	1.22	**0.049**	1.06	0.091
Antihypertensives	Never used	Ref		Ref		Ref		Ref		Ref		Ref	
	Continuous use	0.06	0.934	-0.40	0.599	-0.31	0.679	-0.25	0.469	-0.40	0.273	-0.36	0.334
	Started use after Phase 1	-1.65	**0.024**	-1.72	**0.021**	-1.90	**0.011**	-0.97	**0.006***	-0.97	**0.007***	-0.96	**0.009***
	Stopped use after Phase	0.35	0.761	-0.47	0.696	-0.43	0.720	-0.47	0.394	-0.50	0.390	-0.46	0.425
Diabetes medications	Never used	Ref		Ref		Ref		Ref		Ref		Ref	
	Continuous use	1.10	0.192	3.09	0.053	2.92	0.067	0.48	0.234	0.61	0.428	0.41	0.596
	Started use after Phase 1	0.28	0.744	0.63	0.535	0.61	0.548	0.07	0.862	0.09	0.849	-0.02	0.963
	Stopped use after Phase 1	-1.51	0.710	-0.35	0.941	-2.29	0.633	-0.08	0.967	0.08	0.974	-0.35	0.881
NSAID analgesics	Never used	Ref		Ref		Ref		Ref		Ref		Ref	
	Continuous use	-1.36	0.293	-1.83	0.169	-1.68	0.200	-0.52	0.393	-0.59	0.355	-0.53	0.406
	Started use after Phase 1	0.49	0.568	0.48	0.576	0.44	0.610	0.14	0.734	0.12	0.777	0.16	0.709
	Stopped use after Phase 1	1.57	0.074	1.66	0.062	1.59	0.071	0.35	0.412	0.34	0.431	0.33	0.439

HorvathAA: DNAm HorvathAge acceleration; HannumAA: DNAm HannumAge acceleration; GrimAA: DNAm GrimAge acceleration; PhenoAA: DNAm PhenoAge acceleration.

Longitudinal models include subjects that had DNA methylation measured at both Phases 1 and 2. Participants whose smoking status changed between Phase 1 and Phase 2 were removed.

Model 1: Change in DNA methylation age acceleration (Phase 2-Phase 1) ~ change in medication use + phase 1 covariates (age, DNAm age acceleration, sex, BMI, smoking, alcohol, education)

Model 2: Model 1 + phase 1 covariates (hypertension, stroke, coronary heart disease, diabetes, HDL, Triglyceride, LDL)

Model 3: Model 2 + blood cell proportions
Medication use variables: diuretics, calcium channel blockers, antihypertensives, diabetes medications, and NSAID analgesics
Beta is the regression coefficient of the respective variable from the regression model, as stated below.
Bold values denote significance at $P < 0.05$. Asterisk denotes Bonferroni corrected significance at $P < 0.05/5= 0.01$.
